# Genetic Engineering of Carbon Monoxide-dependent Hydrogen-producing Machinery in *Parageobacillus thermoglucosidasius*

**DOI:** 10.1264/jsme2.ME20101

**Published:** 2020-10-22

**Authors:** Yuka Adachi, Masao Inoue, Takashi Yoshida, Yoshihiko Sako

**Affiliations:** 1 Graduate School of Agriculture, Kyoto University, Kitashirakawa Oiwake-cho, Sakyo-ku, Kyoto, Kyoto 606–8502, Japan

**Keywords:** hydrogen, carbon monoxide, water-gas shift reaction, markerless gene deletion, *Parageobacillus*

## Abstract

The metabolic engineering of carbon monoxide (CO) oxidizers has the potential to create efficient biocatalysts to produce hydrogen and other valuable chemicals. We herein applied markerless gene deletion to CO dehydrogenase/energy-converting hydrogenase (CODH/ECH) in the thermophilic facultative anaerobe, *Parageobacillus thermoglucosidasius*. We initially compared the transformation efficiency of two strains, NBRC 107763^T^ and TG4. We then disrupted CODH, ECH, and both enzymes in NBRC 107763^T^. The characterization of growth in all three disruptants under 100% CO demonstrated that both enzymes were essential for CO-dependent growth with hydrogen production in *P. thermoglucosidasius*. The present results will become a platform for the further metabolic engineering of this organism.

Hydrogenogenic carbon monoxide (CO) oxidizers are microbes that conserve energy by coupling CO oxidation with hydrogen (H_2_) production in the following reaction (WGSR; water-gas shift reaction): CO+H_2_O↔CO_2_+H_2_ (Fox *et al.*, 1996; Fukuyama *et al.*, 2020). In WGSR, CO is oxidized by a CO dehydrogenase (CODH), which is the only enzyme that oxidizes CO (Xavier *et al.*, 2018), and electrons are transferred to an energy-converting hydrogenase (ECH), in‍ ‍which protons and sodium ions are translocated (Schoelmerich and Müller, 2019). These microbes have the potential to produce H_2_, an energy source with a low environmental impact, on an industrial scale from industrial waste gases containing CO (Fukuyama *et al.*, 2020).

A well-known fermenter, *Parageobacillus thermoglucosidasius* has been identified as a hydrogenogenic CO oxidizer, the genome of which contains one *codh* gene cluster comprising three genes: *cooCSF* for a mature CODH enzyme (AOT13_RS13425–13415 on the NCBI RefSeq genome, NZ_CP012712) and one *ech* gene cluster that comprises twelve genes for a mature ECH complex (AOT13_RS13410–13355) (Fig. 1A), both of which are required for the formation of an active CODH/ECH supercomplex (Mohr *et al.*, 2018a). Among hydrogenogenic CO oxidizers, *P. thermoglucosidasius* is the first reported organism that is a thermophilic facultative anaerobe. The type strain DSM 2542^T^ is capable of growing under a mixture of‍ ‍both CO and O_2_ by initially consuming O_2_ via aerobic respiration, followed by CO by WGSR (Mohr *et al.*, 2018a; 2018b). Another strain, TG4, is capable of growing even under a 100% CO atmosphere (Inoue *et al.*, 2019b). Regarding industrial applications, thermophiles are ideal because of higher H_2_ production rates, cost-effectiveness in temperature control, and lower contamination risks in cultivation at higher temperatures (Diender *et al.*, 2015). Moreover, facultative anaerobes are preferable for industrial applications because they do not require any specialized methods to maintain anoxic culture conditions (Lee *et al.*, 2019). Under the above-described criteria, *P. thermoglucosidasius* is an ideal organism for the applications.

Genetic engineering is regarded as one of the major approaches to establish metabolically manipulated cell lines with the more efficient biocatalytic production of H_2_ from CO (Fukuyama *et al.*, 2020). Genetic engineering methods and tools have been developed in *P. thermoglucosidasius* to delete target genes (Cripps *et al.*, 2009; Bacon *et al.*, 2017) and transform exogenous genes (Taylor *et al.*, 2008; Reeve *et al.*, 2016). However, previously developed genetic engineering methods have not been applied to its CO-dependent H_2_-producing machinery. It is important to note that the gene-deleted strains developed to date in other hydrogenogenic CO oxidizers are not marker-free (Kerby *et al.*, 1992; 1997; Kim *et al.*, 2013). In contrast, markerless gene deletions of the gene cluster for CO-dependent H_2_ production allow subsequent deletions at multiple loci or the heterologous overexpression of CODH/ECH and other metabolic enzymes with the same selection marker to achieve higher H_2_ production rates and generate other valuable chemicals.

In the present study, the markerless deletions of *codh*, *ech*, and *codh*–*ech* in *P. thermoglucosidasius* NBRC 107763^T^, hereafter called the Δ*codh*, Δ*ech*, and Δ*codh*–*ech* strains, respectively, were successfully performed. We then characterized the phenotypes of the disruptants under 100% CO conditions. This is the first study to have established markerless disruptions of CODH and ECH in hydrogenogenic CO oxidizers.

The strains NBRC 107763^T^ (=DSM 2542^T^) (Suzuki *et al.*, 1983) and TG4 (Inoue *et al.*, 2019b) were used in the present study. Both strains were routinely cultured under aerobic conditions in liquid TGP medium containing the following (L^–1^): 17‍ ‍g tryptone, 3‍ ‍g soypeptone, 4‍ ‍mL glycerol, 4‍ ‍g sodium pyruvate, 5‍ ‍g NaCl, and 2.5‍ ‍g K_2_HPO_4_ (Cripps *et al.*, 2009). Regarding gene disruption, pUC18-derived suicide plasmids (pUC18K-CODHdel, pUC18K-ECHdel, pUC18K-CODHECHdel) were constructed, which contained a kanamycin resistance gene (*kan^R^*) and three homology arms, *i.e.*, upstream (‘up’), downstream (‘down’), and 5′ (‘start’), for each target gene in the following sequence: 5′-‘start’-*kan^R^*-‘up’-‘down’-3′ (Fig. 1B, C, and D). The arrangement was designed for a simple two-step homologous recombination. The plasmids were introduced into NBRC 107763^T^ using a high osmolality transformation method (Taylor *et al.*, 2008). Markerless gene disruptants were obtained using a two-step homologous recombination strategy (Cripps *et al.*, 2009; Bacon *et al.*, 2017) (Fig. 1B, C, and D), in which the first step relied on double crossovers replacing the target gene by the *kan^R^* cassette, and after serially passaging the transformants, the 2nd step excised the *kan^R^* cassette from the genome and was selected by replica plating (Fig. 1B, C, and D). Gene disruptions were confirmed by genomic PCR and whole-genome shotgun sequencing. It is important to note that the putative promoter region of the *codh*-*ech* cluster and the 118-bp intergenic region upstream of *ech* were left in the Δ*codh* and Δ*ech* strains. In the phenotypic characterization of the disruptants under CO, the wild-type strain (WT) and disruptants were cultivated in inorganic modified B medium (Yoneda *et al.*, 2013) supplemented with 0.1% yeast extract under 100% CO conditions at 65°C and 100 rpm using 250-‍mL glass bottles sealed with rubber stoppers and polypropylene screw caps. During the experimental procedure, cell growth was monitored by optical density at 600 nm (OD_600_), and the gas composition was analyzed by gas chromatography. All plasmids and primers used in the present study are listed in Table S1 and Table S2, respectively. Further methodological details are outlined in Supplementary Materials.

To select an appropriate strain for the genetic engineering study, we initially compared the transformation efficiency of a plasmid between the type strain NBRC 107763^T^ and strain TG4, which we had isolated. Three different types of *Escherichia coli*-*Geobacillus* shuttle plasmids, namely, pG1C, pG2K, and pG1AK-PheB (Reeve *et al.*, 2016) (Table S1), were transformed into the two strains. Transformation efficiency was then estimated from average colony-forming units μg^–1^ of DNA. The transformation efficiencies of TG4 ranged between 0 and 5.8, while those for NBRC 107763^T^ were between 1.4×10^4^ and 1.7×10^4^, as reported previously (Reeve *et al.*, 2016) (Table 1). This result may be attributed to putative restriction enzymes uniquely encoded in the TG4 genome (PTHTG4_RS00115 and PTHTG4_RS11255 on NCBI RefSeq genome, NZ_BHZK01000001), which are enzymes that digest exogenous plasmids. Therefore, we hereafter used strain NBRC 107763^T^ in gene deletion experiments.

The markerless gene deletion procedure was performed using *P. thermoglucosidasius* NBRC 107763^T^ to establish Δ*codh*, Δ*ech*, and Δ*codh–ech* strains (Fig. 1), according to Bacon *et al.* (2017), with modifications in plasmids, cultivation conditions, the number of passages, and the selection marker (Supplementary Methods). Each of the knockout plasmids (3‍ ‍μg) was transformed into NBRC 107763^T^ and an average of six transformant colonies were obtained for each plasmid. After the colonies had been serially passaged four times into fresh liquid TGP medium containing kanamycin, the correct insertion of the *kan^R^*-containing plasmid cassette at the first crossover site was confirmed by the length of the PCR products using appropriate primer sets (data not shown). In *codh* and *codh*–*ech* gene disruptions, colonies with the first crossover occurring at both ‘start’ and ‘down’ were selected (Fig. 1B and C). Δ*codh* and Δ*codh*–*ech* with the second crossover at ‘up’ were obtained by replica plating using the 11th and 20th passages in liquid TGP medium without kanamycin. In contrast, in *ech* gene disruption, no colonies were obtained with the first crossover occurring at both ‘start’ and ‘down’ even though we additionally passaged the transformants 30 times. Therefore, colonies with the first crossover occurring only at ‘down’ were selected, and Δ*ech* with the second crossover occurring at ‘up’ was then obtained after passaging 16 times in medium without kanamycin (Fig. 1D). Genomic PCR clearly indicated that the genes were successfully deleted at the appropriate positions without leaving markers (Fig. 1E). We also performed whole-genome shotgun sequencing of all four strains including WT, and mapped the sequenced reads onto the DSM 2542^T^ complete genome sequence (NCBI RefSeq genome, NZ_CP012712) to support gene deletions at the appropriate sites, to confirm no marker insertion into the genomes, and to identify unexpected gene mutations. Consistent with the PCR results, no reads were mapped onto the loci of *codh*, *ech*, and *codh*–*ech* in Δ*codh*, Δ*ech*, and Δ*codh*–*ech*, respectively (Fig. S1). Moreover, no reads were mapped onto the *kan^R^* and plasmid backbone sequences, indicating the success of markerless gene deletions (data not shown). In comparisons with the reference genome of DSM 2542^T^, six mutations were detected in WT (Table S4). In comparisons with the parental strain WT, there were three, nine, and five missense or nonsense mutations in the protein-coding genes in Δ*codh*, Δ*ech*, and Δ*codh*–*ech*, respectively (Table S5). However, no significant differences were observed in growth or morphology under aerobic conditions in liquid modified B medium supplemented with 0.4% glucose between WT and the disruptants (rod-shaped) (data not shown). Furthermore, Δ*codh* and Δ*ech* had no mutations in *ech* and *codh*, respectively, and none of the disruptants had mutations in peripheral protein-coding genes (0.3‍ ‍Mbp around *codh*–*ech*) (Table S5 and S6). It is important to note that in comparisons with WT, no common mutations were detected in the three disruptants (Table S5), suggesting that the common phenotypes of the disruptants under CO (see below) were due to deletions in our target genes, *codh* and *ech*.

We also examined the growth of the disruptants and WT under 100% CO gas conditions (Fig. 2). Anaerobically grown cells were inoculated into fresh liquid medium as the OD_600_ reached 0.001. All strains grew with similar growth rates until the OD_600_ reached 0.03 in the first 3 h, and then decreased to 0.02 in the next 10 h. The OD_600_ of WT was maintained at approximately 0.02 for the next approximately 30 h, and WT then launched CO-dependent growth via WGSR at 40 h. WT growth had a doubling time of 6.9 h until the OD_600_ reached 0.25, converting all available CO to H_2_ and CO_2_ by 118 h (Fig. 2A). In contrast, the OD_600_ of the disruptants decreased to approximately 0.01 after the first 13‍ ‍h, while slight fluctuations in the OD_600_ were observed in Δ*ech* and Δ*codh*–*ech* only after approximately 40–80 h for unknown reasons (Fig. 2B, C, and D). In all disruptants, neither H_2_ nor CO_2_ was produced even after a 120-h cultivation (Fig. 2B, C, and D). In contrast to rod-shaped cells in WT at 50 h of cultivation, irregularly shaped cells, *i.e.*, lysed, elongated, and rounded cells, were observed in Δ*codh*, Δ*ech*, and Δ*codh*–*ech* at the same time point (data not shown). The growth rates and OD_600_ of WT and the disruptants showed similar changes in the first 13 h (Fig. 2). They may have used yeast extracts in the medium for fermentation-mediated energy production prior to CO-dependent growth. This result indicates that the disruptants grew even under 100% CO when other substrates, such as yeast extract, were available. In contrast, CO-dependent growth with the production of H_2_ and CO_2_ was completely abolished in all disruptants, which was consistent with CO being an energy source for the WT of *P. thermoglucosidasius*, and this also indicated that CODH and ECH are essential for CO-dependent H_2_ production. Although the expression of downstream *ech* may be impaired by a polar effect in Δ*codh*, CODH is considered to be responsible for CO-dependent growth and H_2_ production activity because it is widely accepted that ECH does not oxidize CO (Schoelmerich and Müller, 2019) and CODH is the only enzyme that oxidizes CO in *P. thermoglucosidasius* (Mohr *et al.*, 2018a). In addition, similar findings on abolishment were reported by Kerby *et al.* (1995), with *cooS* being disrupted by the *kan^R^* insertion in *Rhodospirillum rubrum*, a mesophilic, hydrogenogenic CO oxidizer. The present results showing that Δ*codh* and Δ*ech* abolished both H_2_-producing and CO-oxidizing abilities also indicate that CODH and ECH are mutually and exclusively dependent on each other for CO-dependent H_2_ production in *P. thermoglucosidasius*.

Our markerless disruptants allow additional gene manipulations with the same marker kanamycin, enabling the further engineering of the CO-dependent H_2_-producing machinery in *P. thermoglucosidasius*. H_2_ may be produced more efficiently by the heterologous expression of O_2_-tolerant *Carboxydothermus hydrogenoformans* CODH-IV (Domnik *et al.*, 2017) or other types of CODHs (Inoue *et al.*, 2019a) in Δ*codh*. CO-independent H_2_ production may also become possible if ECH is coupled to other pathways, by introducing genes for certain enzymes, such as pyruvate-ferredoxin oxidoreductase, which provide electrons for the proton reduction of ECH via ferredoxin, as demonstrated in *Clostridia* spp. (Abo-Hashesh *et al.*, 2011). Similarly, valuable chemicals may be produced using CO from waste gas by connecting CODH to other metabolic pathways in Δ*ech*. The present results may open new avenues in the industrial application of facultative anaerobic thermophiles with the ability for hydrogenogenic CO oxidation to the production of H_2_, which will act as a renewable and sustainable source of clean energy for future generations.

## Accession number

Raw data for whole-genome shotgun sequencing have been deposited in the DNA Data Bank of Japan Sequence Read Archive (DRA010757).

## Citation

Adachi, Y., Inoue, M., Yoshida, T., and Sako, Y. (2020) Genetic Engineering of Carbon Monoxide-dependent Hydrogen-producing Machinery in *Parageobacillus thermoglucosidasius*. *Microbes Environ ***35**: ME20101.

https://doi.org/10.1264/jsme2.ME20101

## Supplementary Material

Supplementary Material

## Figures and Tables

**Fig. 1.**
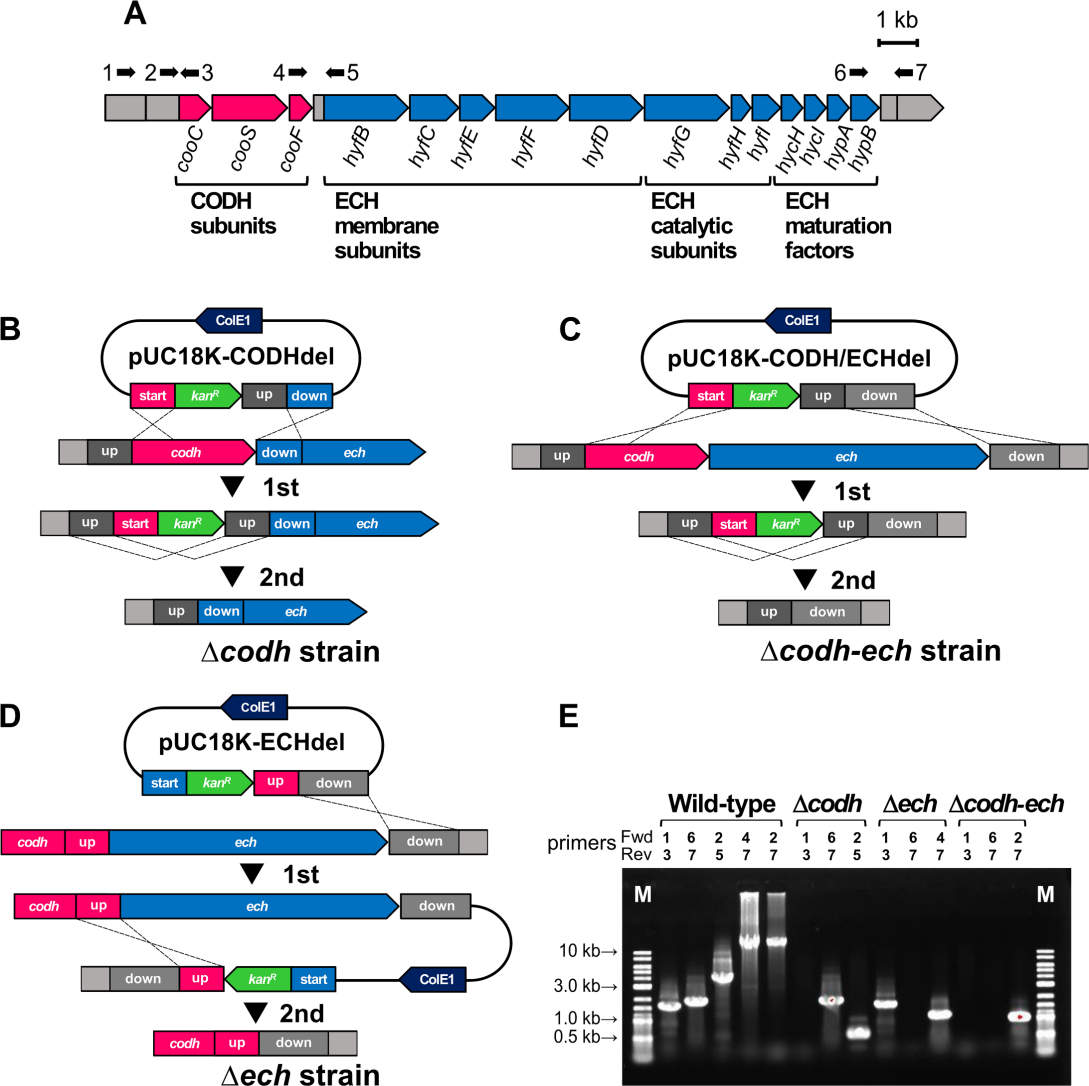
CODH/ECH gene deletion in *Parageobacillus thermoglucosidasius*. (A) A schematic representation of the CODH/ECH gene cluster in *P. thermoglucosidasius*. The arrows with numbers indicate the annealing sites and directions of PCR amplification. (B–D) Schematic representations of markerless gene deletion strategies used in the strains Δ*codh* (B), Δ*codh-ech* (C), and Δ*ech* (D). The three words ‘start’, ‘up’, and ‘down’ represent the 5′-end of the target genes, upstream of the target genes, and downstream of the target genes, respectively. In Δ*codh* and Δ*codh*–*ech*, the first crossovers occurred at ‘start’ and ‘down,’ and the second crossovers occurred at ‘up’ (B, C). In Δ*ech*, the first crossover occurred at ‘down’ only and the second crossover occurred at ‘up’ (D). (E) Confirmation of gene deletions by genomic PCR. Agarose gel electrophoresis of PCR products is shown. Primer numbers and annealing sites are designated as arrows in (A).

**Fig. 2.**
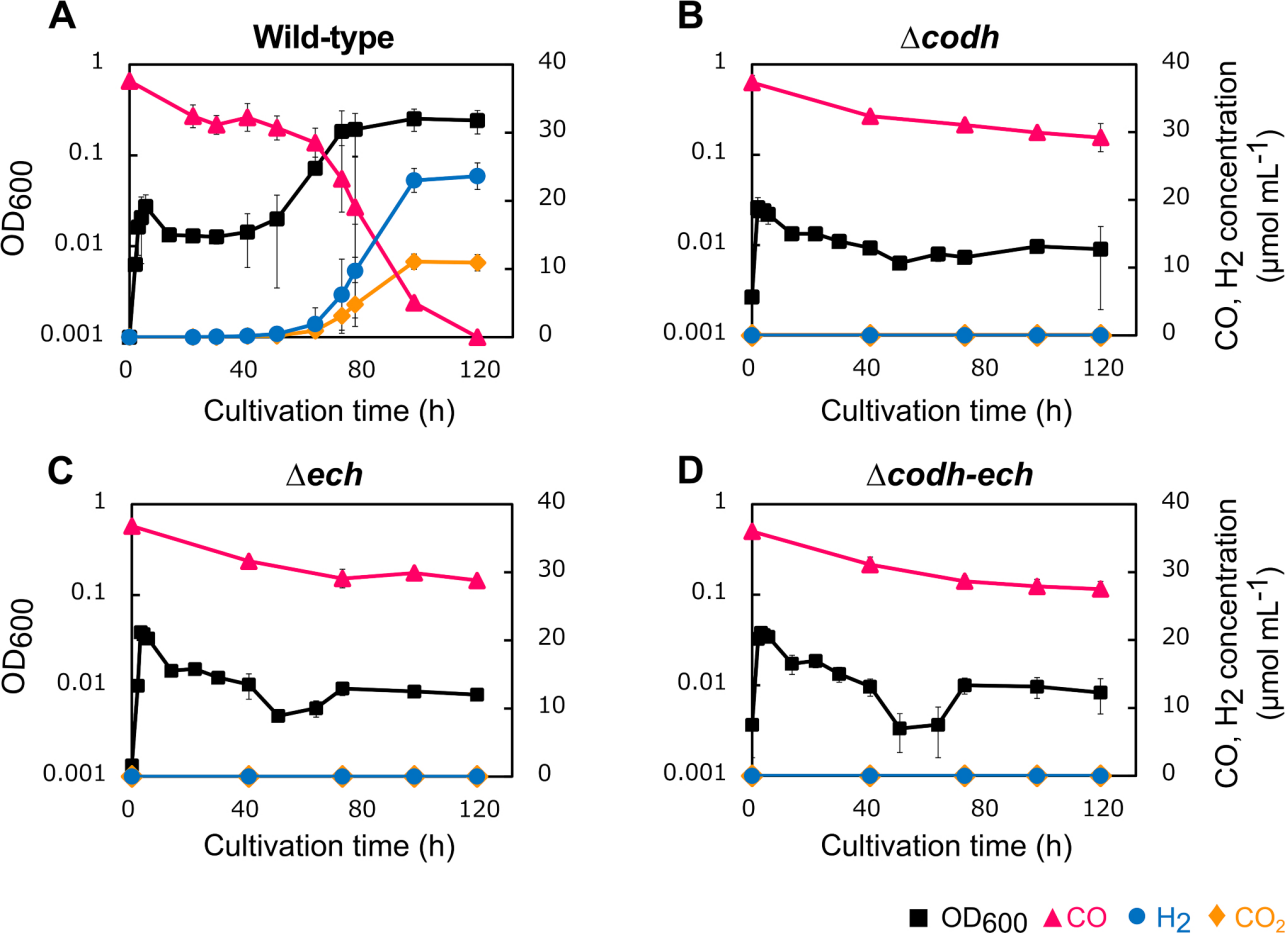
Phenotypic characterization under a CO atmosphere in wild-type (A), Δ*codh* (B), Δ*ech* (C), and Δ*codh-ech* (D) strains. Growth, CO consumption, and H_2_ production were monitored during the cultivation under 100% CO at 65°C. The left vertical axis shows OD_600_ (black square). The right vertical axis shows concentrations of CO (pink triangle), CO_2_ (orange diamond), and H_2_ (blue circle) in the gas phase. Error bars indicate standard deviations (*n*=3).

**Table 1.** Transformation efficiency of strains NBRC107763^T^ and TG4

Strain	Plasmid	Transformation efficiency^a^
NBRC 107763^T^	pG1C	1.7×10^4^±1.3×10^4^
	pG2K	1.5×10^4^±0.8×10^4^
	pG1AK-PheB	1.4×10^4^±1.1×10^4^
TG4	pG1C	0
	pG2K	5.8±4.4
	pG1AK-PheB	5.4±5.1

^a^ Mean value of colony-forming units μg^–1^ DNA with the standard error of the mean. There were three biological replicates in NBRC 107763^T^ and four in TG4.
