# Supplementary material for: Genetic Engineering of Carbon Monoxide-dependent Hydrogen-producing Machinery in Parageobacillus thermoglucosidasius
Source: Microbes Environ. 2020 Oct 22;35(4):ME20101. doi: 10.1264/jsme2.ME20101 (PMC7734403; doi:10.1264/jsme2.ME20101)
Supplement: Supplementary file 1 — Supplementary Material [file 35_20101_s1.pdf]

Supplementary Material

Genetic Engineering of Carbon Monoxide-Dependent Hydrogen-Producing Machinery in *Parageobacillus thermoglucosidasius*

Yuka Adachi, Masao Inoue, Takashi Yoshida, Yoshihiko Sako

Graduate School of Agriculture, Kyoto University, Kitashirakawa Oiwake-cho, Sakyo-ku, Kyoto 606-8502, Japan

- Supplementary Methods
- Supplementary Figure
- Supplementary Tables
- Supplementary References

Supplementary Methods

Strains and Growth Conditions

Parageobacillus thermoglucosidasius type strain NBRC 107763^T was purchased from the Biological Resource Center, National Institute of Technology and Evaluation (NBRC) (Chiba, Japan). The strain TG4 was isolated from a marine sediment in Kagoshima Bay, Japan and maintained in our laboratory (Inoue *et al.*, 2019). *P. thermoglucosidasius* strains were routinely cultured in TGP medium (Cripps *et al.*, 2009). *Escherichia coli* NEB10β was purchased from New England Biolabs (NEB) (Beverly, MA, USA) and cultured in Luria-Bertani medium. When required, the following antibiotics were added to the medium: 12 μg/mL kanamycin and 10 μg/mL chloramphenicol for *P. thermoglucosidasius*, and 50 μg/mL kanamycin for *E. coli*. For culture experiment, B medium (Yoneda *et al.*, 2013) was modified to contain 0.05% NH_4Cl, 0.01% KH_2PO_4, 0.02% $MgCl_2 \cdot 6H_2O$, 0.01% $CaCl_2 \cdot 2H_2O$ supplemented with 0.1% yeast extract, and NaCl was excluded. Cultivation was performed under 100% CO at 65°C, 100 rpm in 100 mL of the modified B medium using 250-mL serum bottles sealed with rubber stoppers and a polypropylene screw cap. Cell growth was monitored by measuring optical density at 600 nm (OD_{600}) using Ultrospec 2100 pro (Biochrom, Berlin Germany). Gas composition was analyzed by a GC-2014 gas chromatography system (Shimadzu, Kyoto, Japan) equipped with a thermal conductivity detector and a Shincarbon ST packed column (Shinwa Chemical Industries, Kyoto, Japan), using argon as carrier gas.

Transformation

For transformation, a high osmolarity electroporation method (Taylor *et al.*, 2008) was used. Three *E. coli-Geobacillus* shuttle plasmids, pG1C, pG2K, and pG1AK-PheB

(Reeve *et al.*, 2016) (Table S1), were transformed into the strains NBRC 107763^T and TG4. The cells were spread onto TGP agar medium containing kanamycin or chloramphenicol and cultured overnight at 52°C. Transformation efficiency was estimated from average colony forming units per microgram of DNA using three and four biological replicates for NBRC 107763^T and TG4, respectively.

Plasmid Constructions

For gene deletions, knockout plasmids were constructed by assembling PCR-amplified fragments using NEBuilder HiFi DNA Assembly (NEB). All plasmids and primers used in the present study are listed in Table S1 and Table S2, respectively. The *codh* knockout plasmid, pUC18K-CODHdel, consisted of five fragments: pUC18 (generated from pG2K using the primers 23 and 24), 5′-end of *cooCSF* (‘start’) (600 bp; generated from genomic DNA using the primers 31 and 32), kanamycin resistance gene (*kan*^{R}) (generated from pG2K using the primers 27 and 28), upstream of *cooCSF* (‘up’) (600 bp; generated from genomic DNA using primers 25 and 26), and downstream of *cooCSF* (‘down’) (600 bp; generated from genomic DNA using primers 29 and 30). It was a suicide plasmid as *Geobacillus* replication origin (repB) was not included in the PCR fragment. The other two knockout plasmids, pUC18K-ECHdel and pUC18K-CODH/ECHdel, were constructed in a similar manner with the following modifications: the fragment size of downstream of *ech* was changed to 1,200 bp to increase the chances of recombination, and pUC18 replication origin (ColE1, 840 bp) was PCR-amplified using the primers 37 and 38, instead of amplifying pUC18 (1,184 bp) to facilitate the assembly.

Gene disruptions

codh, *ech*, and *codh–ech* were deleted in NBRC 107763^T based on a markerless gene deletion method following the previously adopted strategy (Bacon *et al.*, 2017, Cripps *et al.*, 2009) (Fig. 1). The strategy was based on a two-step homologous recombination. The first step relied on double homologous recombination where the target gene was replaced by kan^R. The second step relied on single homologous recombination which pinched off kan^R and other plasmid inserts from the genome. It should be noted that, since double crossovers did not occur in the first step, Δ*ech* was generated by changing the strategy using two steps. The first step was based on single homologous recombination where the whole plasmid was inserted into the genome. The second step relied on single homologous recombination which pinched off plasmid sequence and the target genes. Our strategy may be one of the simplest strategies for markerless gene deletion, which only involves serial passaging and replica plating methods. NBRC 107763^T was transformed by 3 μg of each knockout plasmid and grown overnight on TGP plates containing kanamycin at 52°C. All the colonies were cultured and serially passaged four times in fresh liquid TGP medium containing kanamycin, to increase the chances of recombination, and subsequently grown overnight on TGP agar medium. Correct insertion of the kan^R-containing plasmid cassette at the first crossover site were then checked in the resulting transformants by the length of the PCR products using appropriate primers by colony PCR. Then, appropriate transformants were selected and grown in the liquid TGP medium without kanamycin at 65°C for 6 h until kan^R dropped off from the genomes by second crossovers. The resulting transformants were screened for kanamycin resistance by replica plating onto TGP plates with and without kanamycin. Finally, marker-free gene deletions were confirmed by genomic PCR and whole genome

shotgun sequencing.

Genome Sequencing and Analysis

Genomic DNA of the strains WT, Δ*codh*, Δ*ech*, and Δ*codh–ech* was extracted using the DNeasy blood and tissue kit (Qiagen, Hilden, Germany), according to the manufacturer's instructions. A DNA library was prepared using the Nextera XT DNA Library Preparation Kit (Illumina, San Diego, CA), according to the manufacturer's instructions. Sequencing was performed by the Illumina MiSeq platform, which generated total 14,638,574 paired-end reads (Table S4). Quality trimming and adapter removal were performed using Fastp version 0.20.1 (Chen *et al.*, 2018), with the following settings: sliding window with a window size of 4 bases and with an average Phred quality score >Q30, >95% of bases with Phred quality score of >Q30, and minimum length of 50 bases. After these procedures, over 2 million reads in each strain remained. The read processing and mapping results are summarized in Table S3. The filtered reads were mapped onto the complete genome sequence (NZ_CP012712) of DSM 2542^T using BWA version 0.7.17 (Li & Durbin, 2010) with default settings. The mapping result was viewed using Integrative Genomics Viewer (IGV) (Robinson *et al.*, 2011) (Fig. S1). Single-nucleotide polymorphisms and small insertions and deletions were identified using breseq version 0.35.0 (Deatherage & Barrick, 2014) with default settings. The mutations detected by breseq were checked manually on the mapping results obtained by BWA and listed if the mutations occurred in more than 5% reads (Table S4-S6).

Supplementary Figure

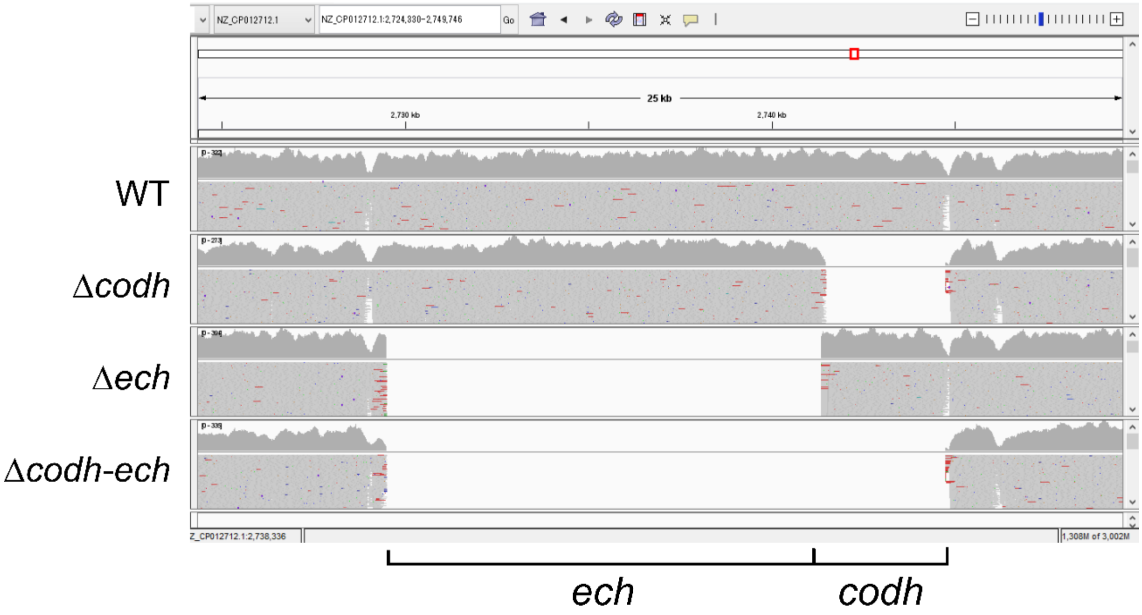

Fig. S1 The genome mapping analysis of the strain WT, Δ*codh*, Δ*ech*, and Δ*codh–ech* in the *codh-ech* genomic region. The reads were obtained by whole genome shotgun sequencing and mapped onto the reference sequence of DSM 2542^{T} (NZ_CP012712) using BWA. The results were shown on IGV in the order of WT, Δ*codh*, Δ*ech*, and Δ*codh–ech* from the top. The read coverage was ~×250 on average in the region. No reads were mapped onto *codh*, *ech*, and *codh–ech* in the strains Δ*codh*, Δ*ech*, and Δ*codh–ech*, respectively, suggesting the genes were knocked out properly in the disruptants.

Supplementary Tables

Table S1 Plasmids used in the present study

Plasmid names	References
pG2K	Reeve *et. al*., 2016
pG1C	Reeve *et. al*., 2016
pG1AK-PheB	Reeve *et. al*., 2016
pUC18K-CODHdel	this study
pUC18K-ECHdel	this study
pUC18K-CODH/ECHdel	this study

Table S2 Primers used in the present study

Nos.	Names	Sequences (5′ to 3′)
1	UP_UP_C_F	CTTAAACAAAAGGGGAACTT
2	inUP_C_F	CTCATAGGGAAGCATAAACT
3	CooC_R	CTTTCTCTATCATCTCGCAA
4	inUP_E_F	GATATAGACGAGAAGAAGGTT
5	inDOWN_C_R	ACTTATCGCTGAAACATAGT
6	inEch_R	CGAGAAAGTAATGATGGTTG
7	inUP_E_R	CATATTCATTTAGCTCCCCT
8	Kan_F	ACGAAGATTAGATGCTATAATTG
9	Kan_R	CTTATCAAAATGGTATGCGT
10	Kan_OUT_F	CGCATACCATTTTGATAAGG
11	Kan_OUT_R	CAATTATAGCATCTAATCTTCGT
12	pUC18_OUT_F	AAACTTGGTCTGACAGTTAC
13	pUC18_OUT_R	AGCTATGACCATGATTACAT
14	inCODH_R	GTTATTGCATCCAACAAAGA
15	inKan_R	AAGGATGGTAGAAATGTTGT
16	inpUC18_F	AATACGGTTATCCACAGAAT
17	inpUC18_R	AACTCTTTTTCCGAAGGTAA
18	ColE1_OUT_F	GGATCTTCACCTAGATCCTT
19	ColE1_OUT_R	CTGTGGATAACCGTATTACC
20	DOWN_E_F	TCGGATAAGATGCAATCA
21	inDOWN_E_F	TTTTAAAAGAGGTGGTAGGA
22	inDOWN_E_R	CGAGCATATTTGCGATGATT

23	A_pUC18_F1	AAATGTAATCATGGTCATAGCTGTTTCC
24	A_pUC18_R1	GCATTGGTAACTGTCAGACCAAG
25	A_UP_C_F	aacttggtctgacagttaccaatgcCATTGTAAGAAGAGGCATCCTTCG
26	A_UP_C_R	ctaatcttcgtttTTAAACGCCTCCTTTGTTCGG
27	A_Kan_F1	aggaggcgtttaaAAACGAAGATTAGATGCTATAATTGTTATTAAAAGGATTGAAG
28	A_Kan_R1	ttgcgcaaaactgCCTTATCAAAATGGTATGCGTTTTGACAC
29	A_DOWN_C_F	cattttgataaggCAGTTTTGCGCAAGGGCG
30	A_DOWN_C_R	aacagctatgaccatgattacatttATTGCTTCCTCATCCTGTTTGAC
31	A_start_C_F	aacttggtctgacagttaccaatgcATGAAATTAGCTATTTCTGGGAAAGGTG
32	A_start_C_R	ctaatcttcgtttTTCTTCATCTTTAGGATGGCGC
33	A_pUC18_F2	tggcagaattgctAAATGTAATCATGGTCATAGCTGTTTCC
34	A_pUC18_R2	gcatcttatccgaTTAAACGCCTCCTTTGTTCG
35	A_DOWN_E_F1	ggaggcgtttaaTCGGATAAGATGCAATCATCGC
36	A_DOWN_E_R1	atgattacatttAGCAATTCTGCCAACACTTC
37	A_colE1_F	ttggcagaattgctAACTCAAAGGCGGTAATACGG
38	A_colE1_R	tgctgaacattcatTGATTTAAAACTTCATTTTTAATTTAAAAGGATCTAGGTG
39	A_start_E_F	gaagttttaaatcaATGAATGTTCAGCAGCTATTTCTGC
40	A_start_E_R	tctaatcttcgtttAACGCTGCGAAAATCAGTAAAG
41	A_kan_F2	attttcgcagcgttAAACGAAGATTAGATGCTATAATTGTTATTAAAAGGATTG
42	A_kan_R2	gaaaatctctttccCCTTATCAAAATGGTATGCGTTTTGAC
43	A_UP_E_R	ccattttgataaggGGAAAGAGATTTTCATTCGTTATGAACG
44	A_UP_E_R	tgcatcttatccgaTTATTCCTACCTCCCGCC
45	A_DOWN_E_F2	ggaggtaggaataaTCGGATAAGATGCAATCATCGC
46	A_DOWN_E_R2	accgcctttgagttAGCAATTCTGCCAACACTTC

*The capital letters in the primers 25-46 indicate annealing nucleotides, and the small letters indicate the overlaps for plasmid assemblies.

Table S3 The read processing and the mapping results

	WT	Δ*codh*	Δ*ech*	Δ*codh-ech*
Total paired-end reads sequenced	3,818,168	2,784,264	4,397,447	3,638,695
Total paired-end reads after quality filtering	3,054,454	2,146,303	3,744,811	2,884,146
Total mapping efficiency (%) (BWA)	95.5	96.4	96.6	97.1
Mean coverage (BWA)	×260	×190	×327	×261

Table S4 Mutation detected in the WT in comparison with the reference genome

Positions	Locus Tags	Genes	Mutations	Annotations
649,737	AOT13_RS03255	peptide MFS transporter	A→G	V429A (GTA→GCA)
729,186	AOT13_RS03625	DNA-directed RNA polymerase subunit delta	C→T	A106V (GCG→GTG)
1,094,858	AOT13_RS05365	helix-turn-helix transcriptional regulator	G→A	W213* (TGG→TAG)
1,736,805	AOT13_RS08685	YggS family pyridoxal phosphate-dependent enzyme	T→C	pseudogene (247/678 nt)
1,861,428	AOT13_RS09295	ribosome maturation factor RimP	(A)$_{8\to7}$	pseudogene (416/475 nt)
2,620,284	intergenic	GNAT family N-acetyltransferase/IS256 family transposase	(C)$_{7\to8}$	intergenic (-167/+73)

Table S5 Missense and nonsense mutations detected in the disruptants

Positions	Locus Tags	Genes	Mutations	Annotations	Δ*codh*	Δ*ech*	Δ*codh-ech*
Δ*codh* and Δ*ech* specific mutations							
1,094,858	AOT13_RS05365	helix-turn-helix transcriptional regulator	G→A	W213* (TGG→TAG)	SNP[a]	SNP	
1,841,155	AOT13_RS09205	chemotaxis response regulator protein-glutamate methylesterase	(A)$_{7\to8}$	coding (493/1059 nt)	INS[b]	INS	
Δ*codh*-specific mutations							
547,052	AOT13_RS02745	RNA-binding protein S1	G→A	Q100* (CAA→TAA)	SNP		
Δ*ech*-specific mutations							
294,278	AOT13_RS01460	exopolysaccharide biosynthesis polyprenyl glycosylphosphotransferase	(T)$_{10\to9}$	coding (17/675 nt)		DEL[c]	
857,147	AOT13_RS04225	histidine kinase	T→C	H109R (CAT→CGT)		SNP	
1,537,195	AOT13_RS07680	hypothetical protein	(A)$_{8\to7}$	coding (584/588 nt)		DEL	
2,464,207	AOT13_RS12105	Rieske 2Fe-2S domain-containing protein	(A)$_{8\to7}$	coding (382/429 nt)		DEL	
3,145,220	AOT13_RS15365	glycerol-3-phosphate responsive antiterminator	G→A	P79S (CCG→TCG)		SNP	
3,150,207	AOT13_RS15405	YppE family protein	T→C	M120V (ATG→GTG)		SNP	
3,255,062	AOT13_RS15985	c-type cytochrome biogenesis protein CcsB	(A)$_{8\to9}$	coding (61/1191 nt)		INS	
Δ*codh-ech*-specific mutations							
229,550	AOT13_RS01210	response regulator transcription factor	Δ1 bp	coding (399/681 nt)			DEL
233,252	AOT13_RS01230	undecaprenyl/decaprenyl-phosphate alpha-N-acetylglucosaminyl 1-phosphate transferase	G→A	S305L (TCG→TTG)			INS
766,532	AOT13_RS03835	NADH-quinone oxidoreductase subunit C	Δ34 bp	coding (415-448/1152 nt)			DEL
914,923	AOT13_RS04580	hypothetical protein	(A)$_{7\to8}$	coding (128/210 nt)			INS
1,565,389	AOT13_RS07835	RNA polymerase sigma factor SigI	C→T	Q35* (CAA→TAA)			SNP

[a]SNP, single nucleotide polymorphism. [b]INS, insertion. [c]DEL, deletion.

Table S6 Mutations without amino acid replacements

Positions	Locus Tags	Genes	Mutations	Annotations	Δ*codh*	Δ*ech*	Δ*codh-ech*
2,305,441	AOT13_RS11350	sodium:solute symporter	C→T	L167L (CTC→CTT)	SNP		
1,160,436	intergenic	hypothetical protein	T→C	intergenic (-453/-226)		SNP	
1,907,967	AOT13_RS09520	site-specific DNA-methyltransferase	G→A	A44A (GCG→GCA)		SNP	
2,842,428	intergenic	flavodoxin family protein/alpha/beta fold hydrolase	C→T	intergenic (+41/+115)			SNP
3,858,867	AOT13_RS19050	N-acetylmuramoyl-L-alanine amidase	C→T	T184T (ACG→ACA)			SNP

[a]SNP, single nucleotide polymorphism.

References

Bacon, L. F., Hamley-Bennett, C., Danson, M. J. & Leak, D. J. (2017) Development of an efficient technique for gene deletion and allelic exchange in *Geobacillus* spp. *Microb. Cell Fact.* **16**(1), 58.

Chen, S., Zhou, Y., Chen, Y. & Gu, J. (2018) Fastp: An ultra-fast all-in-one FASTQ preprocessor. *Bioinformatics*. **34**, i884–i890.

Cripps, R. E., Eley, K., Leak, D. J., Rudd, B., Taylor, M., Todd, M., *et al*. (2009) Metabolic engineering of *Geobacillus thermoglucosidasius* for high yield ethanol production. *Metab. Eng.* **11**(6), 398–408.

Deatherage, D. E. & Barrick, J. E. (2014) Identification of mutations in laboratory-evolved microbes from next-generation sequencing data using breseq. *Methods Mol. Biol.* **1151**, 165–188.

Inoue, M., Tanimura, A., Ogami, Y., Hino, T., Okunishi, S., Maeda, H., *et al*. (2019) Draft genome sequence of *Parageobacillus thermoglucosidasius* strain TG4, a hydrogenogenic carboxydotrophic bacterium isolated from a marine sediment . *Microbiol. Resour. Announc*. **8**(5), e01666–18.

Li, H. & Durbin, R. (2010) Fast and accurate long-read alignment with Burrows-Wheeler transform. *Bioinformatics* **26**(5), 589–595.

Reeve, B., Martinez-Klimova, E., Jonghe, J. de, Leak, D. J. & Ellis, T. (2016) The *Geobacillus* plasmid set: A modular toolkit for thermophile engineering. *ACS Synth. Biol.* **5**(12), 1342–1347.

Robinson, J. T., Thorvaldsdóttir, H., Winckler, W., Guttman, M., Lander, E. S., Getz, G., *et al*. (2011) Integrative genomics viewer. *Nat. Biotechnol.* **29**(1), 24–26.

Taylor, M. P., Esteban, C. D. & Leak, D. J. (2008) Development of a versatile shuttle vector for gene expression in *Geobacillus* spp. *Plasmid* **60**(1), 45–52.

Yoneda, Y., Yoshida, T., Yasuda, H., Imada, C. & Sako, Y. (2013) A thermophilic, hydrogenogenic and carboxydotrophic bacterium, *Calderihabitans maritimus* gen. nov., sp. nov., from a marine sediment core of an undersea caldera. *Int. J. Syst. Evol. Microbiol.* **63**(PART10), 3602–3608.
